# General HIV-Related Knowledge and Attitudes Among Nurses and Nursing Students: A Systematic Review and Meta-Analysis with Narrative Synthesis of Clinical Practices (2011–2026)

**DOI:** 10.3390/healthcare14193159

**Published:** 2026-09-23

**Authors:** Roza Alekesheva, Dzhyldyz Ibraimova, Shnara Svetlanova, Zhanar Ibraimzhanova, Ainur Seitmanova, Galiya Bilibayeva, Sholpan Karzhaubayeva, Indira Karibayeva

**Affiliations:** 1 Department of Nursing, Faculty of Medicine and Public Health, Al-Farabi Kazakh National University, Almaty 050038, Kazakhstan; alekesheva@kaznu.kz (R.A.); ibraimzhanova.zh@kaznu.kz (Z.I.); seitmanova@kaznu.kz (A.S.); bilibayeva@kaznu.kz (G.B.); 2 Department of Public Health and Health Care, Kyrgyz-Russian Slavic University Named After B.N. Yeltsin, Bishkek 720000, Kyrgyzstan; djyldyz.ibraimova70@gmail.com; 3 Department of Nursing, Kazakhstan Medical University “KSPH”, Almaty 050060, Kazakhstan; svetlanova.sh@kaznmu.kz; 4 Salidat Kairbekova National Scientific Center for Healthcare Development, Ministry of Healthcare of the Republic of Kazakhstan, Almaty 050010, Kazakhstan; sh.karzhaubayeva@nrchd.kz; 5 Department of Research Management, JSC Research Institute of Cardiology and Internal Diseases, Almaty 050012, Kazakhstan; 6 Jiann-Ping Hsu College of Public Health, Georgia Southern University, Statesboro, GA 30458, USA

**Keywords:** HIV, nurses, nursing students, knowledge and attitudes, clinical practice

## Abstract

**Highlights:**

**What are the main findings?**

- Across 34 studies, pooled estimates for high/good HIV-related knowledge (52.4%), mean knowledge score (66.3% of the possible score), positive/favorable attitudes (41.6%), and mean favorable-attitude score (48.1% of the possible range) were derived from highly heterogeneous evidence (I^2^ = 97.5–99.8%).

**What are the implications of the main findings?**

- The pooled estimates should be interpreted as descriptive averages across heterogeneous studies rather than as global nursing-workforce benchmarks.
- Future research should use standardized knowledge and attitude instruments and objective or audited measures of HIV-related clinical practice.

**Abstract:**

**Background/Objectives:** Nurses and nursing students are central to HIV prevention and care, but studies of their general HIV-related knowledge, attitudes, and practices use heterogeneous populations, instruments, scoring systems, and thresholds. This review had three objectives: (1) to meta-analyze, separately, the prevalence of study-defined high/good general HIV knowledge, mean general knowledge scores, prevalence of study-defined positive/favorable general attitudes, and mean favorable-attitude scores; (2) to synthesize HIV-related clinical-practice findings narratively when measures were not comparable; and (3) to explore whether pooled knowledge and attitude estimates differed between practicing nurses and nursing students. General outcomes excluded narrowly focused PrEP-, PEP-, stigma-only, and item-level constructs. **Methods:** PubMed/MEDLINE, Scopus, PsycINFO, and CINAHL Complete were searched for eligible quantitative studies. Four knowledge and attitude outcomes were meta-analyzed in separate random-effects models; practice outcomes were not pooled. Heterogeneity, 95% prediction intervals, leave-one-out influence, possible small-study effects, subgroup differences, and certainty of evidence were assessed. **Results:** Thirty-four studies were included. The pooled prevalence of high/good HIV-related knowledge was 52.4% (95% CI 30.4–73.5%; I^2^ = 97.5%; 95% prediction interval 3.6–97.0%), and the pooled mean knowledge score was 66.3% of the possible score (95% CI 60.8–71.8%; I^2^ = 99.8%; prediction interval 40.0–92.5%). Positive/favorable attitudes had a pooled prevalence of 41.6% (95% CI 7.3–86.5%; I^2^ = 99.1%; prediction interval 0.0–99.9%), and the pooled mean favorable-attitude score was 48.1% of the possible range (95% CI 41.9–54.2%; I^2^ = 99.7%; prediction interval 21.3–74.8%). Thus, all four meta-analyses showed extreme heterogeneity and wide prediction intervals. Prespecified subgroup tests did not detect nurse-versus-student differences for either knowledge outcome or favorable-attitude prevalence; a higher pooled mean favorable-attitude score among students was observed in one exploratory comparison. Four studies reported non-equivalent practice indicators (16.0–85.3%), which were not pooled. Certainty was very low for all meta-analyzed outcomes. **Conclusions:** The pooled estimates should be interpreted as descriptive summaries across heterogeneous operationalizations rather than as precise global nursing-workforce benchmarks or common competency thresholds. More standardized measurement, objective assessment of clinical practice, and longitudinal and interventional research are needed.

## 1. Introduction

Globally, the age-standardized HIV prevalence rate reached 499.7 per 100,000 population in 2023 (95% uncertainty interval: 474.8–524.3) [1]. An estimated 1.2 million new infections occurred in 2025, bringing the total number of people living with HIV (PLHIV) to 40.9 million [2]. While 88% of PLHIV globally now know their status, this falls short of the 95% target set for 2025, leaving an estimated 5.3 million people undiagnosed and disconnected from care [2]. Closing this gap depends heavily on the frontline health workforce—and nurses, as the largest cadre of health professionals and frequently the first point of clinical contact for PLHIV, occupy a structurally critical position in HIV testing, counselling, treatment initiation, and ongoing care [3,4].

What nurses know, believe, and do in relation to HIV is not incidental to outcomes; it is directly tied to them. Nurses’ knowledge shapes the accuracy of risk counseling and the appropriateness of referrals for prevention technologies such as pre-exposure and post-exposure prophylaxis (PrEP/PEP) [5]. Their attitudes influence whether people living with HIV (PLHIV) experience clinical encounters as safe or stigmatizing [6]. Their daily practices determine whether guideline-based care is consistently delivered at the point of contact. Stigma—whether enacted or anticipated—within healthcare settings has been repeatedly identified as a structural barrier slowing progress toward the global HIV testing and treatment cascade [7]. Surveys show that a significant proportion of young PLHIV avoid health facilities specifically because of their HIV status [8]. When nurses themselves hold stigmatizing attitudes or lack applied clinical knowledge, this barrier is not abstract; it operates directly at the clinical interface where testing, linkage to care, and prevention counseling should occur.

In this review, the three KAP domains were treated as related but distinct constructs rather than as a single composite outcome. General HIV-related knowledge comprised broad understanding of HIV transmission, prevention, treatment, and care as operationalized by eligible study instruments. General attitudes comprised overall favorable or unfavorable evaluations of HIV and people living with HIV; studies restricted to stigma, discrimination, willingness, or a single attitudinal item were not treated as equivalent to a general attitude measure. Clinical practices comprised reported behaviors relevant to HIV care or prevention. Although knowledge can support informed attitudes and practice, the review did not assume a simple causal sequence from knowledge to attitudes to behavior, because practice also depends on professional role, experience, institutional context, and opportunity.

Practicing nurses and nursing students were both included because they represent consecutive stages of the nursing workforce: students reflect the pre-registration training environment, whereas practicing nurses reflect knowledge and attitudes after clinical exposure and continuing professional education. The subgroup comparison was intended to explore whether population type accounted for part of the between-study variation, not to infer an effect of professional status. Aggregate comparisons may be confounded by age, professional and life experience, country, curriculum, instrument, study year, and clinical setting; they were therefore treated as exploratory.

Individual studies assessing nurses’ knowledge, attitudes, and practices (KAP) toward PLHIV have accumulated steadily over the past decade and a half, across diverse countries and clinical contexts. However, this evidence base remains fragmented in three specific ways. First, syntheses in adjacent domains—for example, KAP studies of healthcare workers toward other stigmatized or emerging infectious conditions—have repeatedly shown that pooled estimates of “good knowledge” or “favorable attitudes” can diverge sharply from individual study claims once heterogeneity in instruments and populations is accounted for [9]. Yet, to our knowledge, no comparable quantitative synthesis has been conducted specifically for nurses’ and nursing students’ HIV-related KAP. Second, existing HIV-specific syntheses tend to focus on a single domain in isolation—for instance, broader healthcare-worker PrEP knowledge and willingness, or HIV-related stigma and its general correlates—rather than jointly quantifying knowledge, attitudes, stigma, and practices within the same workforce [6,10,11]. This limits the ability to compare relative deficits across domains or to determine whether knowledge gaps and stigma move together or independently within nursing populations. The contemporary literature remains difficult to interpret because studies have used different populations, instruments, scale directions, scoring conventions, and study-defined thresholds for “adequate,” “good,” or “favorable” outcomes. Consequently, it is unclear whether variation in reported findings reflects genuine differences between settings or differences in measurement.

This uncertainty is particularly significant now: global HIV financing has been severely disrupted since early 2025, with reductions in international assistance already limiting access to prevention and care services in several high-burden regions [12,13]. These disruptions increase the relative importance of the existing health workforce, and nurses within it, in sustaining HIV testing, prevention counseling, and stigma-free care delivery without corresponding increases in external support.

Accordingly, this review had three objectives. First, to meta-analyze four distinct general HIV-related outcomes reported from 2011 onward: (i) prevalence of study-defined high/good knowledge; (ii) mean knowledge score expressed as a percentage of the possible score range; (iii) prevalence of study-defined positive/favorable attitudes; and (iv) mean favorable-attitude score expressed on a 0–100 percentage-of-possible-range scale. Second, to synthesize HIV-related clinical-practice findings narratively when measures were not sufficiently comparable for pooling. Third, to test, in prespecified exploratory subgroup analyses, whether each pooled knowledge or attitude outcome differed between practicing nurses and nursing students. Knowledge, attitude, and practice outcomes were not combined into a single KAP meta-analysis.

## 2. Materials and Methods

### 2.1. Study Design

This systematic review was conducted using prespecified evidence-synthesis methods informed by JBI guidance for systematic-review conduct and critical appraisal and by Cochrane guidance for quantitative synthesis, unit-of-analysis decisions, and interpretation of heterogeneous evidence. PRISMA 2020 was used as the reporting guideline [14]. The review was registered in PROSPERO (CRD420261433354).

The review commenced on 1 April 2026. The initial database searches were conducted on 8 May 2026, and the PROSPERO record was submitted and registered on 25 June 2026. Because searching preceded registration, the chronology is reported explicitly. At the time of PROSPERO submission, database searching had been completed, but formal data extraction and quantitative synthesis had not commenced. Registration therefore preceded data extraction and analysis, although it did not precede the literature search.

All stages of the systematic review process, including database searching, title and abstract screening, full-text eligibility assessment, data extraction, and risk of bias appraisal, were conducted independently by two trained reviewers. Disagreements at any stage were resolved through discussion; where consensus could not be reached, a third independent reviewer adjudicated [15].

### 2.2. Eligibility Criteria

Eligibility criteria were defined prospectively using the Population, Intervention/Exposure, Comparator, Outcome, and Study design (PICOS) framework.

Population (P): Eligible populations included practicing or qualified nurses, including registered nurses, licensed or enrolled nurses, charge nurses, advanced-practice nurses, and midwives, as well as students enrolled in pre-registration or undergraduate nursing programs. Nursing students were included because they represent the incoming nursing workforce and because knowledge and attitudes developed during pre-registration education may influence subsequent clinical care. Because practicing nurses and nursing students differ in clinical experience and professional responsibilities, results were extracted separately and compared in prespecified subgroup analyses. Mixed healthcare-provider studies were eligible only when nurse-specific data could be extracted. Studies reporting exclusively on physicians, pharmacists, or other non-nursing healthcare professionals with no extractable nurse-specific data were excluded.

Intervention/Exposure (I): Studies were eligible when they reported at least one quantitative measure of general HIV-related knowledge, general attitudes toward HIV or people living with HIV, or HIV-related clinical practice. Studies focusing specifically on PrEP, PEP, mother-to-child transmission, occupational exposure, needle-stick injury prevention, or another narrowly defined HIV topic were excluded unless a separately extractable general HIV-knowledge or general HIV-attitude outcome was reported. Studies reporting only stigma, discrimination, avoidance, willingness, or an individual attitudinal item without an eligible general knowledge or attitude outcome were excluded. Stigma subscales and item-level discriminatory-intent measures were not pooled as independent outcomes.

Comparator (C): No comparator was required.

Outcomes (O): Four outcomes were eligible for quantitative synthesis: (i) the proportion classified as having high/good general HIV-related knowledge; (ii) the mean general HIV-knowledge score transformed to a 0–100 scale; (iii) the proportion classified as having a positive/favorable general attitude toward HIV or people living with HIV; and (iv) the mean general attitude score transformed to a 0–100 scale and oriented so that higher values indicated more favorable attitudes. HIV-related clinical-practice outcomes were extracted but were quantitatively pooled only when the underlying behaviors, scoring approaches, and definitions were sufficiently comparable.

Study design (S): Eligible quantitative designs were those capable of contributing a cross-sectional estimate of an eligible outcome, including descriptive or analytical cross-sectional studies, cohort or case–control studies when eligible baseline or cross-sectional data could be extracted, and randomized or non-randomized intervention studies when pre-intervention baseline data were available. Only baseline data were used from intervention studies; intervention effects and post-intervention outcomes were excluded. When multiple baseline groups were reported, eligible groups were combined at the study level so that each study contributed no more than one estimate per outcome. Thus, eligibility was based on the nature of the data contributed to this review rather than on pooling intervention effects across different study designs. Qualitative studies, editorials, letters, conference abstracts without full-text data, and grey literature were excluded. Studies published in languages other than English were also excluded.

### 2.3. Search Strategy

PubMed/MEDLINE, Scopus, PsycINFO, and CINAHL Complete were searched. The search was restricted to studies published from January 2011 onward because the present review was designed to update the evidence published following the previous 2011 systematic review of trained nurses’ HIV-related knowledge, attitudes, and practices [16]. Accordingly, the review concerns contemporary post-2010 evidence and does not represent a synthesis of the complete historical literature. Searches were limited to English-language, peer-reviewed studies involving human participants.

Search strategies were developed by the review team in consultation with a medical librarian and were tailored to the controlled vocabulary and indexing system of each database. Three conceptual blocks were used across all databases: (1) nursing workforce terms (e.g., nurse, nursing student, midwife, nurse practitioner); (2) HIV/AIDS terms (e.g., HIV, AIDS, human immunodeficiency virus); and (3) KAP domain terms (e.g., knowledge, attitude, practice, willingness, preparedness, competency). Controlled vocabulary was used in PubMed, PsycINFO, and CINAHL Complete, together with database-specific free-text fields. Scopus was searched using structured title, abstract, and keyword terms. The complete search strategies for each database are presented in Table 1.

In addition to electronic database searching, the reference lists of all included studies and relevant systematic reviews identified during screening were hand-searched to identify additional eligible studies. No search of grey literature, trial registries, or unpublished data was conducted. After the revision, CINAHL Complete was searched again in July 2026 to ensure the completeness of the database search.

### 2.4. Study Selection

All records retrieved from the four databases were imported into Rayyan (Qatar Computing Research Institute), an online systematic review management platform, for deduplication and screening. Duplicate records were identified and removed automatically, with manual verification. Following deduplication, two reviewers independently screened all titles and abstracts against the pre-specified eligibility criteria. Records that were clearly irrelevant were excluded; those that could not be excluded with certainty based on title and abstract alone were retained for full-text assessment.

Both reviewers independently assessed the full text of all retained records. The eligibility decision and reason for exclusion were documented for each record. Disagreements were resolved through discussion; if consensus was not reached, a third reviewer provided a binding adjudication.

### 2.5. Data Extraction

Data were extracted using a standardized template developed a priori in Microsoft Excel, piloted before use, and documented in the PROSPERO protocol. Two reviewers independently extracted data from all included studies; discrepancies were resolved by consensus or third-reviewer adjudication. The following data were extracted from each study: (1) Study characteristics: first author, year of publication, country, WHO region, World Bank income classification, study design. (2) Participant characteristics: total sample size and population type (registered nurses, nursing students, advanced practice nurses, or midwives). (3) Knowledge outcomes: assessment instrument and item content, maximum possible score, reported mean and standard deviation (SD) of the knowledge score, and the number and proportion of participants classified as having adequate or good knowledge. (4) Attitude outcomes: assessment instrument, scoring direction (higher score = more favorable vs. higher score = more stigmatizing), maximum possible score, reported mean and SD, and the number and proportion of participants with positive or favorable attitudes. (5) Practice outcomes: assessment instrument, practice domains covered, and the number and proportion of participants demonstrating good or adequate practice.

Continuous knowledge and attitude scores were transformed to a percentage of the theoretically possible score range. For scales with theoretical minimum L, theoretical maximum U, and reported mean M, the percentage of the possible scale range was calculated as [(M − L)/(U − L)] × 100. For knowledge instruments with a minimum of zero, this simplifies to (M/U) × 100. For attitude instruments in which higher scores represented less favorable or more stigmatizing attitudes, the transformed score was calculated as 100 − {[(M − L)/(U − L)] × 100}. The theoretical range of each instrument was used.

Rescaling standardized only the numeric range and direction of continuous scores; it did not make instruments equivalent in item content, construct coverage, reliability, or validity. Likewise, categorical prevalence outcomes retained each study’s own threshold, so “high/good” and “positive/favorable” categories were not assumed to be psychometrically identical across studies. Pooling therefore represents a pragmatic construct-level synthesis, and the resulting values were interpreted as descriptive averages across heterogeneous operationalizations.

Where studies reported only frequencies without denominators, or where denominators differed across variables, variable-specific denominators were used. Each study contributed no more than one independent estimate to each meta-analysis. For intervention studies with multiple baseline groups, proportion numerators and denominators were summed across groups. Continuous baseline means and standard deviations were combined using standard study-level aggregation formulas. Item-level or subscale estimates were not entered as separate independent observations.

### 2.6. Risk of Bias Assessment

Because the meta-analyses synthesized descriptive study-level estimates rather than intervention effects, risk of bias was appraised according to the type of evidence contributed to the review rather than the nominal design of the parent publication. All contributing study-level datasets, including pre-intervention baseline observations from randomized and non-randomized intervention studies, were evaluated using the JBI Critical Appraisal Checklist for Studies Reporting Prevalence Data. The nine checklist items assess the appropriateness of the sample frame, participant sampling, sample-size adequacy, description of participants and setting, coverage of the identified sample, validity of outcome measurement, consistency and reliability of measurement, appropriateness of statistical analysis, and response rate/non-response management. For continuous knowledge and attitude outcomes, the measurement items were interpreted in relation to the validity and consistent application of the relevant score instrument. Each item was judged as Yes, No, Unclear, or Not applicable. Item-level judgments were reported without converting them to a numerical total score or an arbitrary low/moderate/high category. Two reviewers independently completed the reappraisal; disagreements were resolved by consensus or third-reviewer adjudication.

### 2.7. Description of Outcome Measures

Four knowledge and attitude outcomes were quantitatively synthesized in separate meta-analyses; no composite KAP outcome was constructed.

Outcome 1: Prevalence of high/good HIV-related knowledge. The primary numerator included participants meeting the highest study-defined knowledge category. Participants classified only as moderate or satisfactory were excluded from the primary numerator. A separate supplementary analysis (Supplementary Figure S1) used the broader study-defined threshold of moderate/satisfactory-or-higher knowledge where such categories were available; this was distinct from the primary high/good outcome and was not used as the principal knowledge estimate.

Outcome 2: Mean HIV-related knowledge score. Continuous scores were transformed to a 0–100 percentage-of-possible-range scale.

Outcome 3: Prevalence of positive/favorable attitudes. The numerator included participants meeting the study-defined criterion for a positive or favorable general HIV-related attitude.

Outcome 4: Mean favorable-attitude score. Continuous general attitude scores were transformed to a 0–100 scale and oriented so that higher values consistently indicated more favorable attitudes.

Clinical-practice findings were treated as a separate outcome domain and synthesized narratively because eligible studies assessed substantively different behaviors and used non-equivalent definitions and instruments. Practice outcomes were not pooled with knowledge or attitude outcomes and were not treated as a fifth meta-analyzed outcome.

### 2.8. Statistical Analysis and Meta-Analysis Plan

All meta-analyses were conducted using the meta and metafor packages in R, version 4.5.1 (R Foundation for Statistical Computing, Vienna, Austria). Each of the four knowledge or attitude outcomes was pooled in a separate random-effects model; knowledge, attitudes, and clinical practices were never combined in a single pooled model. Proportion outcomes were pooled using logistic-normal random-intercept generalized linear mixed models. Continuous scores transformed to a common 0–100 scale were pooled using inverse-variance random-effects models with restricted maximum-likelihood estimation of between-study variance. Freeman–Tukey double-arcsine models were used only as sensitivity analyses and were not described as GLMMs. The 0–100 transformation standardized numeric range and direction for presentation but did not establish psychometric equivalence among instruments with different content, reliability, validity, or construct coverage. Pooled estimates were reported with 95% confidence intervals and 95% prediction intervals; prediction intervals were used to show the expected range of true effects in a new, similar study. The study, rather than an individual arm, subscale, or item, was the unit of analysis.

Between-study heterogeneity was quantified using the I^2^ statistic, Cochran’s Q test, and the τ^2^ (tau-squared) estimator. I^2^ values were interpreted as: <25% low, 25–50% moderate, 50–75% substantial, and >75% considerable heterogeneity, in accordance with Higgins et al. (2003) [17]. Given the diversity of instruments, populations, and settings across included studies, high heterogeneity was anticipated a priori; therefore, random-effects models were used throughout regardless of I^2^ values.

A prespecified subgroup analysis compared practicing nurses with nursing students separately for each meta-analyzed outcome. Differences between subgroup estimates were evaluated using formal tests for subgroup interaction. These were aggregate study-level comparisons and did not adjust for age, professional or life experience, country, curriculum, instrument, study year, or clinical setting. Because few studies were available within some subgroups and heterogeneity remained substantial, subgroup findings were considered exploratory; a non-significant subgroup test was interpreted as failure to detect a difference rather than evidence of equivalence. A leave-one-out analysis was conducted in which each study was sequentially omitted and the remaining studies re-pooled to identify any single study exerting undue influence on the pooled estimate.

For meta-analytic pools with k ≥ 10 studies, publication bias was assessed using funnel plot visual inspection. For pools with k < 10, formal publication bias testing was not conducted due to insufficient statistical power.

### 2.9. Certainty of Evidence Assessment

The certainty of evidence for each primary meta-analytic outcome was assessed using the Grading of Recommendations Assessment, Development and Evaluation (GRADE) framework [18,19]. GRADE rates the certainty of evidence for each outcome across five domains: (1) risk of bias, (2) inconsistency, (3) indirectness, (4) imprecision, and (5) publication bias (other considerations). The certainty of evidence was rated as High, Moderate, Low, or Very Low.

Because most of the included studies were cross-sectional observational or quasi-experimental in design, the starting certainty for all outcomes was Low (two levels below the High certainty starting point for randomized trials). The certainty was further downgraded if serious concerns were identified in any of the five GRADE domains. Upgrades were considered for large effect sizes, dose–response relationships, or scenarios where all plausible residual confounding would strengthen rather than weaken the conclusion; no upgrades were applied in this review. GRADE assessments were conducted by two reviewers independently.

For the risk of bias domain, a ‘Serious’ downgrade was applied when the majority of contributing studies were rated ‘Moderate concern’ or above on the applicable risk of bias tool. A ‘Very serious’ downgrade was applied when most studies were rated ‘Moderate to high concern’ or when the overall evidence was dominated by studies with critical methodological limitations. For the inconsistency domain, ‘Very serious’ was applied when substantial heterogeneity in instruments, constructs, and populations precluded meaningful interpretation of the pooled estimate, in addition to statistical heterogeneity. For imprecision, a ‘Serious’ downgrade was applied when fewer than five studies contributed to the pool or when the total sample size was below thresholds sufficient for stable proportion or mean estimation.

## 3. Results

### 3.1. Study Selection and Characteristics of the Included Studies

The systematic database search identified 6836 records across four electronic databases: PubMed (*n* = 1989), Scopus (*n* = 3575), PsycINFO (*n* = 822), and CINAHL (*n* = 450). Following the removal of 2465 duplicate records, 4371 records remained for title and abstract screening. Of these, 4152 records were excluded by the reviewers as not meeting the inclusion criteria, leaving 219 reports sought for full-text retrieval. One report could not be retrieved, resulting in 218 reports assessed for eligibility. Full-text review led to the exclusion of 204 reports for the following reasons: focus on patient outcomes not relevant to the review question (*n* = 81), coinfection research (*n* = 31), qualitative study design (*n* = 29), populations comprising non-nursing professionals (*n* = 27), review articles (*n* = 14), study protocols (*n* = 8) [20,21,22,23,24,25,26,27], case studies (*n* = 5) [28,29,30,31,32], and conference abstracts (*n* = 2). Specific PrEP and PEP knowledge studies were excluded [33,34,35,36,37,38,39]. Following additional citation backtracking and an updated CINAHL search, 87 full-text articles were assessed for eligibility. Of these, 32 were excluded for focusing on patient outcomes, 16 for addressing non-nursing healthcare populations, 8 for stigma as the primary focus, 5 for coinfection, 5 for assessing item-level knowledge or attitudes [40,41,42,43,44], and 1 for stigma tool validation [45]. Ultimately, 34 studies met all eligibility criteria and were included in the review: 14 from the initial search and 20 from the additional search. The full study selection process is presented in Figure 1.

The 34 included studies were published between 2011 and 2026 and were conducted across all six WHO regions, with the largest representation from Europe (*n* = 10) and Africa (*n* = 5), followed by South-East Asia, the Americas, the Eastern Mediterranean, and the Western Pacific. Study settings spanned a range of income levels, including high-income (*n* = 11), upper-middle-income (*n* = 13), and lower-middle-income (*n* = 9) countries, with one study reporting mixed-income samples. The majority of studies employed a cross-sectional design (*n* = 27), while the remainder used experimental or quasi-experimental designs, including randomized controlled trials, pre-post educational interventions, and mixed-methods approaches.

Study populations consisted of registered or practicing nurses (*n* = 17) and nursing or nursing/trainee students (*n* = 17), with sample sizes ranging from 28 to 922 participants. A wide variety of validated and adapted instruments were used to assess knowledge, attitudes, and practices, most commonly variants of the HIV Knowledge Questionnaire and the AIDS Attitude Scale, and researcher-developed KAP questionnaires. Detailed characteristics of each included study are presented in Table 2.

Risk-of-bias reappraisal showed that the strongest and most consistently reported domains were description of study participants and settings and appropriateness of statistical analysis, which were judged adequate in all 34 studies. Outcome measurement was also generally consistent: valid measurement methods were judged adequate in 28 studies and unclear in six, while standard/reliable application was judged adequate in 29 studies and unclear in five. The principal concerns related to representativeness and non-response. Participant sampling was judged appropriate in 12 studies, inappropriate in 16, and unclear in six; only eight studies reported sufficient information to judge sample-size adequacy, while this item remained unclear in 26 studies. Response rate or management of non-response was adequate in 16 studies, inadequate in three, and unclear in 15. The sample frame was judged appropriate in 26 studies, inappropriate in seven, and unclear in one. Item-level judgments are presented in Supplementary Table S1.

### 3.2. Outcome-Specific Synthesis Results

#### 3.2.1. Knowledge Outcomes (Separate Meta-Analyses)

Nine studies involving 2115 participants contributed to the meta-analysis of the prevalence of high/good HIV-related knowledge (Figure 2A). Six studies included nurses (*n* = 1954), and three included nursing students (*n* = 161). Among nurses, the reported prevalence ranged from 17% to 84%, with a pooled prevalence of 49.3% (95% CI: 28.0–70.9%). Among nursing students, individual estimates ranged from 9.5% to 86.8%, and the pooled prevalence was 58.3% (95% CI: 10.1–94.5%). The overall pooled prevalence of high/good HIV-related knowledge was 52.4% (95% CI: 30.4–73.5%). Heterogeneity was considerable in both the nurse subgroup (I^2^ = 98%, τ^2^ = 1.3; *p* < 0.0001) and the nursing-student subgroup (I^2^ = 95.7%, τ^2^ = 4.8; *p* < 0.0001), resulting in substantial overall heterogeneity (I^2^ = 97.5%, τ^2^ = 1.9; *p* < 0.0001). The 95% prediction interval ranged from 3.6% to 97.0%, indicating that the prevalence observed in a future comparable study could vary widely across settings. There was no evidence of a difference between nurses and nursing students (χ^2^ = 0.07, df = 1; *p* = 0.791). The forest plot showing the pooled prevalence of moderate/satisfactory and high/good HIV knowledge across 11 study estimates is presented in Supplementary Figure S1. Supplementary Figure S1 uses a broader moderate/satisfactory-or-higher knowledge threshold and is secondary to the primary high/good knowledge outcome; its pooled estimate is therefore not directly comparable with the 52.4% primary estimate.

Nineteen studies involving 5204 participants contributed to the meta-analysis of mean HIV knowledge scores expressed as a percentage of the maximum possible score (Figure 2B). Twelve studies included nurses (*n* = 3605), among whom mean scores ranged from 31.4% to 84.0%. The pooled mean score for nurses was 67.1% (95% CI: 59.4–74.8%). Seven studies included nursing students (*n* = 1599), with mean scores ranging from 49.1% to 79.4% and a pooled mean of 64.7% (95% CI: 57.3–72.1%). The overall pooled mean HIV knowledge score was 66.3% of the maximum possible score (95% CI: 60.8–71.8%). Heterogeneity was extremely high among studies of nurses (I^2^ = 99.9%, τ^2^ = 184.4; *p* < 0.0001), studies of nursing students (I^2^ = 98.1%, τ^2^ = 97.7; *p* < 0.0001), and across all studies (I^2^ = 99.8%, τ^2^ = 147.9; *p* < 0.0001). The corresponding 95% prediction interval was 40.0–92.5%. Mean knowledge scores did not differ significantly between nurses and nursing students (χ^2^ = 0.19, df = 1; *p* = 0.66). Given the broad prediction intervals and very high heterogeneity, the pooled estimates should be interpreted as average values across highly diverse populations, instruments, and study settings.

#### 3.2.2. Attitude Outcomes (Separate Meta-Analyses)

Six studies involving 2154 participants contributed to the meta-analysis of the prevalence of positive/favourable HIV-related attitudes (Figure 3A). Three studies included nurses (*n* = 1343), among whom the prevalence ranged from 0.0% to 54.5%. The pooled prevalence among nurses was 9.7% (95% CI: 0.4–74.8%). Three studies included nursing students (*n* = 811), with individual estimates ranging from 57.1% to 95.7% and a pooled prevalence of 80.3% (95% CI: 43.1–95.7%). The overall pooled prevalence of positive/favourable attitudes was 41.6% (95% CI: 7.3–86.5%). Heterogeneity was considerable among studies of nurses ($I^{2}=98.6\%$, $\tau^{2}=8.0$; p<0.0001), studies of nursing students ($I^{2}=97.4\%$, $\tau^{2}=2.2$; p<0.0001), and across all studies ($I^{2}=99.1\%$, $\tau^{2}=7.2$; p<0.0001). The 95% prediction interval ranged from 0.0% to 99.9%, indicating that the prevalence in a future comparable study could vary across almost the entire possible range. The difference between nurses and nursing students did not reach statistical significance ($\chi^{2}=3.7$, df = 1; p=0.055).

Fifteen studies involving 5095 participants contributed to the meta-analysis of mean favourable HIV attitude scores expressed as a percentage of the possible scale range (Figure 3B). For eligible general HIV-attitude instruments scored in an adverse direction, scores were reverse-coded so that higher standardized values consistently represented more favourable attitudes. Seven studies included nurses (*n* = 2167), among whom mean scores ranged from 34.6% to 47.6%. The pooled mean favourable attitude score among nurses was 41.3% (95% CI: 37.7–45.0%). Eight studies included nursing students (*n* = 2928), with mean scores ranging from 43.3% to 84.1% and a pooled mean of 54.0% (95% CI: 44.5–63.4%). The overall pooled mean favourable attitude score was 48.1% of the possible scale range (95% CI: 41.9–54.2%). Heterogeneity was substantial among studies of nurses ($I^{2}=95.7\%$, $\tau^{2}=28.3$; p<0.0001), studies of nursing students ($I^{2}=99.7\%$, $\tau^{2}=185.0$; p<0.0001), and across all studies ($I^{2}=99.7\%$, $\tau^{2}=146.0$; p<0.0001). The corresponding 95% prediction interval ranged from 21.3% to 74.8%. Nursing students had a significantly higher pooled favourable attitude score than nurses, although this subgroup difference should be interpreted cautiously because of the very high between-study heterogeneity ($\chi^{2}=5.9$, df = 1; p=0.015).

#### 3.2.3. Clinical-Practice Outcomes (Narrative Synthesis)

Four studies involving 1711 participants reported HIV-related clinical-practice indicators (Table 3). Quantitative pooling was not undertaken because the studies assessed substantively different behaviours and used non-equivalent practice definitions. Hassan et al. [46] reported that 147 of 920 nurses (16.0%) did not refuse to provide direct care to a patient with AIDS; this represented a single behaviour rather than an overall practice score. Akhter et al. [66] classified 162 of 190 nurses (85.3%) as demonstrating good overall HIV-related practice, whereas Boakye et al. [70] reported good practice among 128 of 228 nurses (56.1%). Among nursing students, Mutaru et al. [71] found that 215 of 373 participants (57.6%) demonstrated good HIV/AIDS-prevention practice. Because these outcomes differed in construct, population, scoring method, and clinical context, the findings were synthesized descriptively and should not be directly compared as estimates of a common practice outcome.

#### 3.2.4. Heterogeneity, Influence, Small-Study Effects, and Certainty of Evidence

Leave-one-out sensitivity analyses were performed for all four pooled outcomes to assess whether any individual study disproportionately influenced the results (Figure 4). For the prevalence of high/good HIV-related knowledge, the pooled estimate remained relatively stable after sequential omission of each of the nine studies, ranging from 46.9% (95% CI: 26.0–69.0%) after excluding Shah 2015 [58] to 58.8% (95% CI: 37.7–77.1%) after excluding Mohd Nasri 2025 [73], compared with the primary estimate of 52.4% (95% CI: 30.4–73.5%) (Figure 4A). Heterogeneity remained considerable in all analyses, with $I^{2}$ ranging from 96.8% to 97.8% and $\tau^{2}$ from 1.5 to 2.2. The prediction intervals also remained very wide. Thus, no individual study materially changed the overall interpretation of the high/good knowledge prevalence, although substantial between-study variability persisted.

The pooled mean HIV knowledge score was highly stable across the leave-one-out analyses (Figure 4B). After omitting each of the 19 studies in turn, the pooled mean ranged from 65.3% (95% CI: 59.8–70.7%) after excluding Hughes 2011 [47] to 68.2% (95% CI: 64.0–72.4%) after excluding Shahzadi 2017 [61], compared with the primary pooled mean of 66.3% (95% CI: 60.8–71.8%). Heterogeneity remained extremely high in all analyses ($I^{2}=99.4$–99.8%). Exclusion of Shahzadi 2017 [61] produced the largest reduction in between-study variance, decreasing $\tau^{2}$ from 147.9 to 79.2, but did not materially alter the pooled estimate. These findings indicate that the pooled mean knowledge score was not driven by any single study.

For positive/favourable attitude prevalence, the pooled estimate was more sensitive to the exclusion of individual studies (Figure 4C). The leave-one-out estimates ranged from 27.0% (95% CI: 4.5–74.3%) after excluding Pickles 2012 [52] to 63.1% (95% CI: 27.5–88.5%) after excluding Qadir 2025 [74], compared with the primary pooled prevalence of 41.6% (95% CI: 7.3–86.5%). Nevertheless, all estimates remained highly imprecise, heterogeneity remained very high ($I^{2}=97.0$–99.3%), and the prediction intervals covered almost the entire possible prevalence range. The pooled positive-attitude prevalence should therefore be interpreted cautiously because it was based on a small and highly heterogeneous evidence base.

The pooled mean favourable attitude score remained comparatively stable when each of the 15 studies was omitted in turn (Figure 4D). The resulting pooled means ranged from 45.5% (95% CI: 41.8–49.2%) after excluding Akin 2013 [53] to 49.0% (95% CI: 42.7–55.3%) after excluding Boakye 2023 [70], compared with the primary pooled mean of 48.1% (95% CI: 41.9–54.2%). Exclusion of Akin 2013 [53] reduced $\tau^{2}$ from 146.0 to 48.5, indicating that this study contributed substantially to the between-study variability. However, heterogeneity remained extremely high even after its removal ($I^{2}=99.3\%$). Overall, the pooled mean favourable attitude score was robust to the exclusion of individual studies, but the magnitude of heterogeneity remained substantial.

Funnel plots were examined for the two continuous outcomes because each included at least 10 studies (Figure 5). The funnel plot for mean HIV knowledge scores showed visual asymmetry, with several smaller studies distributed away from the pooled estimate and a wider spread of estimates at lower precision (Figure 5A). The funnel plot for mean favourable HIV attitude scores also appeared asymmetric (Figure 5B). Overall, the plots suggest possible small-study effects, but clinical and methodological heterogeneity remains a plausible alternative explanation.

The certainty of evidence was rated very low for all four meta-analysed outcomes (Table 4). For the prevalence of high/good HIV knowledge, based on nine studies, certainty was downgraded because of risk of bias, very substantial heterogeneity ($I^{2}=98.0\%$), and imprecision reflected by the wide confidence interval around the pooled estimate. Publication bias could not be formally assessed because fewer than 10 studies were available. The evidence for the pooled mean HIV knowledge score, based on 19 studies, was also rated very low. The principal concerns were the predominance of cross-sectional, self-reported data, the risk of bias, and extreme inconsistency across studies ($I^{2}\approx 100.0\%$). Possible publication bias or small-study effects were additionally considered because of funnel-plot asymmetry, although interpretation was limited by the very high heterogeneity. For the prevalence of positive/favourable attitudes toward people living with HIV, based on six studies, certainty was downgraded for risk of bias, extreme heterogeneity ($I^{2}=99.0\%$), and serious imprecision, as indicated by the wide confidence interval and prediction interval. Publication bias was not formally assessable because fewer than 10 studies contributed to this outcome. The evidence for the pooled mean favourable attitude score, based on 15 studies, was likewise rated very low. Downgrading reflected methodological limitations in the included studies, extreme inconsistency ($I^{2}\approx 100.0\%$), and imprecision in the pooled estimate. Funnel-plot asymmetry suggested possible publication bias or small-study effects, but this finding should be interpreted cautiously because of the substantial clinical and methodological heterogeneity. Taken together, these downgrades indicate that the true effect for each outcome may differ substantially from the pooled estimates reported in this review, and all findings should be interpreted as hypothesis-generating rather than definitive.

## 4. Discussion

This review addressed three distinct questions: the distribution of general HIV-related knowledge, the distribution of general HIV-related attitudes, and the available evidence on HIV-related clinical practices, with exploratory nurse-versus-student comparisons within the meta-analyzed knowledge and attitude outcomes. Four knowledge and attitude outcomes were meta-analyzed separately, whereas clinical-practice findings were synthesized narratively. The pooled prevalence of high/good knowledge was 52.4%, the pooled mean knowledge score was 66.3% of the possible range, the pooled prevalence of positive/favorable attitudes was 41.6%, and the pooled mean favorable-attitude score was 48.1% of the possible range. These values are not estimates of a single KAP construct or precise global workforce parameters. All four meta-analyses showed extreme heterogeneity, wide prediction intervals, and very-low-certainty evidence.

The prediction intervals are particularly important for interpretation. The estimated true prevalence of high/good knowledge in a new comparable study could plausibly range from 3.6% to 97.0%, while favorable-attitude prevalence could range across almost the entire possible scale. The pooled values therefore summarize the center of highly dispersed study findings rather than defining universal knowledge or attitude levels. Differences in instruments, scale adaptations, languages, scoring direction, study-defined thresholds, countries, curricula, sampling methods, and clinical exposure probably contributed to this variability.

The categorical and continuous outcomes also capture different aspects of the evidence. Categorical estimates depend on study-specific thresholds for “high,” “good,” “positive,” or “favorable,” whereas transformed mean scores preserve more of the original information but still assume that different instruments can be meaningfully represented on a common percentage-of-possible-range scale. Neither approach eliminates the underlying psychometric differences among instruments. Accordingly, comparisons should focus on the consistency, direction, and range of findings rather than on small differences between pooled point estimates.

The pooled high/good knowledge estimate of 52.4% identified in this review supports the range reported for other stigmatized or emerging infectious conditions among healthcare workers, where pooled “good knowledge” prevalence has clustered in the 26–46% range despite active public health attention [77,78]. A previous 2011 systematic review of nurses’ HIV-related knowledge similarly found that nurses’ knowledge level was generally satisfactory, although certain knowledge gaps persisted [16]. A global meta-analysis of mpox knowledge among 27,731 healthcare workers, for instance, found a pooled good-knowledge prevalence of only 26.0% (95% CI 17.8–34.2%) [77], closely paralleling the present review’s HIV knowledge findings. This cross-condition consistency suggests the knowledge gap identified here is not unique to HIV but may reflect a more general limitation in how frontline nursing knowledge is built and reinforced for conditions combining clinical complexity with social stigma. Notably, the included instruments used different content domains and did not apply a common competency threshold. This conceptual heterogeneity means the pooled point estimate should be read as indicating wide variability in knowledge adequacy rather than a single, generalizable deficit.

Prespecified subgroup tests did not detect a statistically significant nurse-versus-student difference for either knowledge outcome. This should not be interpreted as evidence that the groups are equivalent or that professional status has no effect. The comparison was ecological and may be confounded by age, clinical and life experience, country, curriculum, instrument, study year, and clinical setting. Persistent heterogeneity within both subgroups indicates that population type alone did not account for the observed variability in HIV knowledge.

A specific and clinically important gap not addressed in the present study is the HIV-prevention knowledge. To keep the data homogenous, we excluded PrEP and PEP -knowledge related studies. Nevertheless, a 2025 systematic review and meta-analysis of 34 studies across 12 African countries found that healthcare workers’ PrEP awareness was high (85%, 95% CI 75–91%) but PrEP-specific knowledge was poor (18%, 95% CI 4–55%), with attitudes only moderately positive (46%) and willingness to prescribe at 58% [79]. That same review explicitly identified stigma, inadequate training, and health-system constraints as key barriers to PrEP implementation [79]. This distinction between “awareness” and “actionable knowledge” is analytically important: a nurse who recognizes the term PrEP but cannot counsel a patient on its use, dosing, or eligibility offers limited practical prevention benefit. A 2019 US-based review of 18,265 providers modelled this as a cascade in which awareness (68%) and willingness (66%) far outpaced actual consultation (37%) and prescription (24%), with advanced practice nurses showing higher prescribing odds than physicians where empowered to act (OR = 1.51) [80].

Subgroup tests also did not detect a statistically significant nurse-versus-student difference in the prevalence of favorable attitudes. A higher pooled mean favorable-attitude score was observed among nursing students, but this was a single exploratory subgroup finding, was not reproduced in the categorical attitude analysis, and occurred in the presence of extreme heterogeneity. Because the contributing nurse and student studies differed in age distribution, professional and life experience, country, curriculum, instrument, translation, study year, and clinical setting, this result should not be interpreted as evidence that nursing students generally hold more favorable attitudes than practicing nurses.

The broader HIV literature has identified relationships between greater HIV knowledge and lower levels of stigma or discriminatory attitudes [7]. The present review’s co-occurrence of suboptimal knowledge and suboptimal attitude findings at the nursing-workforce level is consistent with this knowledge–stigma relationship, although the predominantly cross-sectional design of the included primary studies precludes causal inference in either direction.

This matters practically because nurse-mediated stigma is not an abstract attitudinal variable. Stigma and discrimination, including within healthcare settings, are explicitly identified in recent appraisals of global progress toward the UNAIDS 95-95-95 testing, treatment, and viral-suppression targets as a persistent structural barrier slowing advancement toward the 2025/2030 targets, with surveys showing that more than 12% of young PLHIV in West and Central Africa report avoiding health centers specifically because of their HIV status [81,82]. Read against this backdrop, the present review’s findings carry particular weight. Some of the included studies were drawn from Africa and the Eastern Mediterranean—regions carrying a disproportionate share of the global HIV burden. Within this context, nurse-level stigma cannot be treated as a peripheral quality-of-care issue. It is a measurable contributor to the structural barriers slowing regional progress toward global HIV elimination targets.

The evidence concerning HIV-related clinical practice was limited and methodologically heterogeneous. Self-reported practice measures in KAP-type surveys are well documented to be vulnerable to social desirability bias, where respondents systematically over-report behaviors perceived as professionally or socially expected, particularly for sensitive or normatively loaded items [83,84]. Objective indicators such as clinical audit, standardized-patient assessment, observed counselling performance, or routinely collected quality-of-care measures would provide more credible evidence of actual practice.

The findings support the need for continued HIV education in both undergraduate nursing curricula and professional development programs [85,86,87]. The absence of a clear knowledge difference between nurses and nursing students suggests that education should not be confined to initial training. Periodic in-service education may be needed to maintain and update general HIV knowledge throughout professional practice.

The findings identify areas requiring further investigation rather than supporting a specific educational intervention. The variability in both knowledge and attitudes indicates that nursing schools and healthcare institutions should first assess locally relevant gaps using validated instruments before selecting or designing educational programs. Intervention studies are needed to determine whether knowledge-focused education, reflective learning, contact-based approaches, case-based teaching, or combinations of these components produce sustained improvements in attitudes and clinical care. The present review cannot establish the comparative effectiveness of these approaches.

Future studies should converge around a smaller set of validated and culturally adaptable instruments. Consistent use of clearly described scale versions, scoring direction, subscales, and thresholds would improve interpretability and allow more meaningful comparisons across settings. Researchers should report both continuous scores and prespecified categorical thresholds where possible, together with sufficient data to calculate effect estimates and measures of variance [88,89].

Practice assessment requires particular improvement. Future studies should distinguish willingness or intention from actual clinical behavior and should prioritize observed practice, clinical audits, standardized patient assessments, routinely collected indicators, or clearly validated practice scales. Where self-reported measures are used, the potential for social desirability bias should be addressed through questionnaire design and analytical sensitivity analyses.

Longitudinal studies and adequately controlled educational interventions are also required [85]. Cross-sectional studies can identify associations but cannot determine whether knowledge and attitudes change over time or whether improvements translate into better clinical practice. Future intervention studies should include sufficiently long follow-up periods, report intervention content in reproducible detail, and assess whether changes in questionnaire scores correspond to changes in care delivery or patient experience.

This review has several limitations. First, the studies used conceptually and psychometrically diverse instruments. Although continuous scores were transformed to a common 0–100 scale and aligned to a favorable direction, this procedure does not make instruments with different content, item weighting, reliability, validity, or construct coverage fully equivalent. Similarly, categorical outcomes relied on study-defined thresholds for “high,” “good,” “adequate,” “positive,” or “favorable,” and these thresholds were not interchangeable. The pooled results therefore represent descriptive averages rather than estimates based on a common diagnostic or competency criterion. Second, practicing nurses and nursing students were both eligible. Subgroup analyses were used to examine population differences, but these aggregate comparisons are vulnerable to confounding by age and life experience at the participant level and by country, study year, instrument, educational system, and clinical setting at the study level; individual-level confounding could not be adjusted using the published aggregate data. The small number of studies in some subgroups also limited statistical power. Third, the review included pre-intervention baseline observations from intervention studies. These data were combined at the study level and treated as cross-sectional observations, but participation in an intervention study may still differ from participation in a conventional population survey. Most outcomes were self-reported and were therefore susceptible to recall and social-desirability bias. Practice outcomes were particularly heterogeneous and could not be pooled. Fourth, all four meta-analyzed outcomes showed extreme statistical heterogeneity that remained largely unexplained after population subgrouping and leave-one-out analysis. Broad prediction intervals indicate that the pooled estimates may have limited applicability to any particular country or institution. Funnel-plot asymmetry may reflect publication or small-study effects, although methodological heterogeneity provides an alternative explanation. Lastly, the review was restricted to English-language, peer-reviewed studies published from 2011 onward. The temporal boundary was selected to update the literature following the previous 2011 review, but relevant earlier studies and publications in other languages were not re-synthesized. Grey literature and unpublished evidence were also excluded, which may have increased the potential for dissemination bias. Finally, database searching began before PROSPERO registration; the review chronology and all protocol deviations are therefore reported transparently.

## 5. Conclusions

This systematic review and meta-analysis of 34 studies found wide variation in general HIV-related knowledge and attitudes among nurses and nursing students. In the primary categorical knowledge analysis, approximately half of participants met study-defined high/good criteria, while favorable-attitude prevalence varied widely across the contributing studies. Continuous scores averaged 66.3% of the possible range for knowledge and 48.1% for favorable attitudes. These pooled values summarize heterogeneous study operationalizations and should not be treated as competency thresholds or global nursing-workforce benchmarks.

Prespecified subgroup tests did not detect nurse-versus-student differences for the two knowledge outcomes or for favorable-attitude prevalence. A higher mean favorable-attitude score among students was observed in one exploratory comparison, but the finding was not replicated for categorical attitudes and may be confounded by differences between the contributing studies. Clinical-practice evidence was too heterogeneous for pooling. Given the extreme heterogeneity, broad prediction intervals, and very low certainty of evidence, the main implications are the need for more standardized measurement and for longitudinal and interventional research using objective or audited measures of clinical practice.

## Figures and Tables

**Figure 1:**
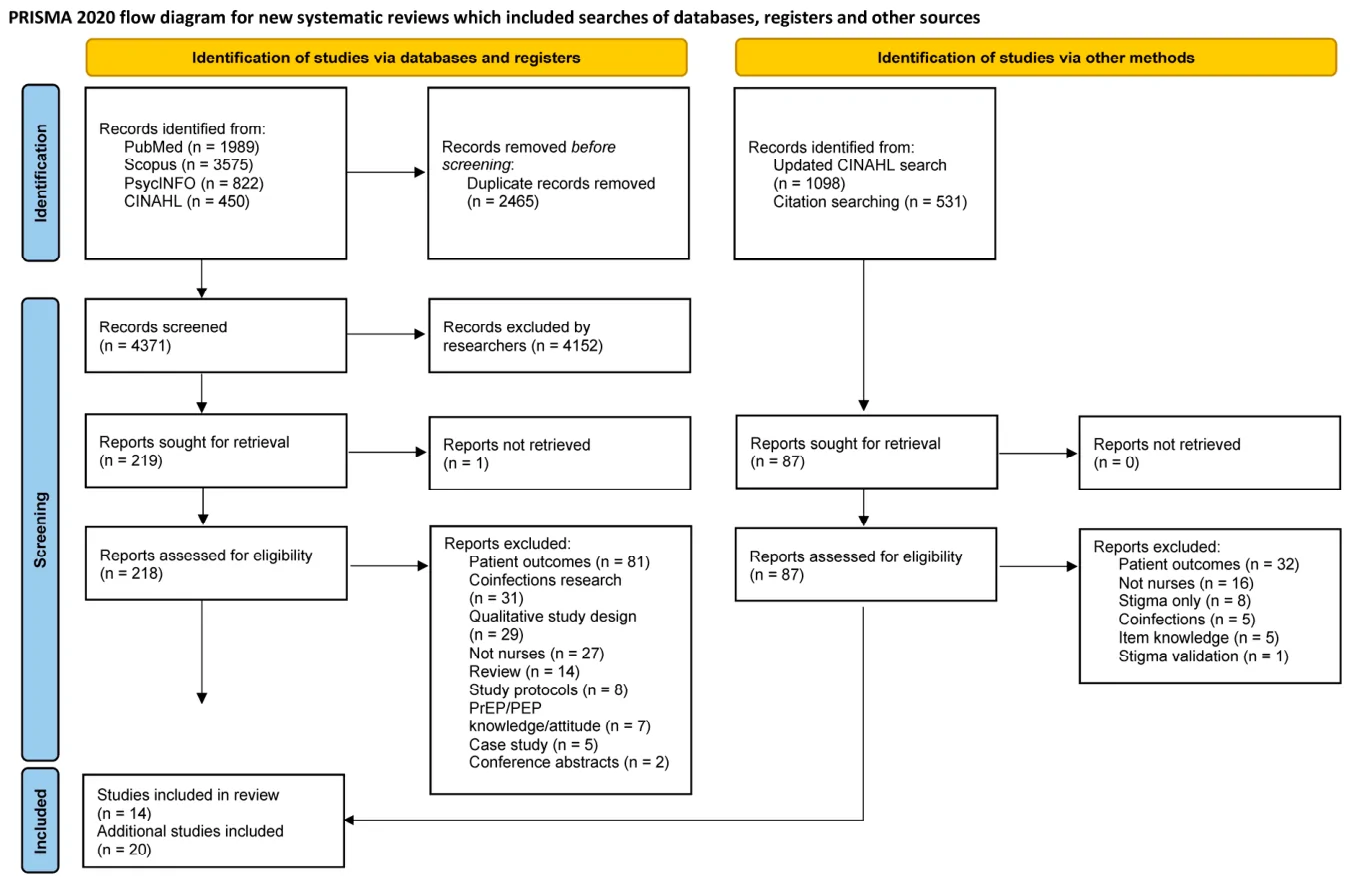
PRISMA 2020 flow diagram of study selection.

**Figure 2:**
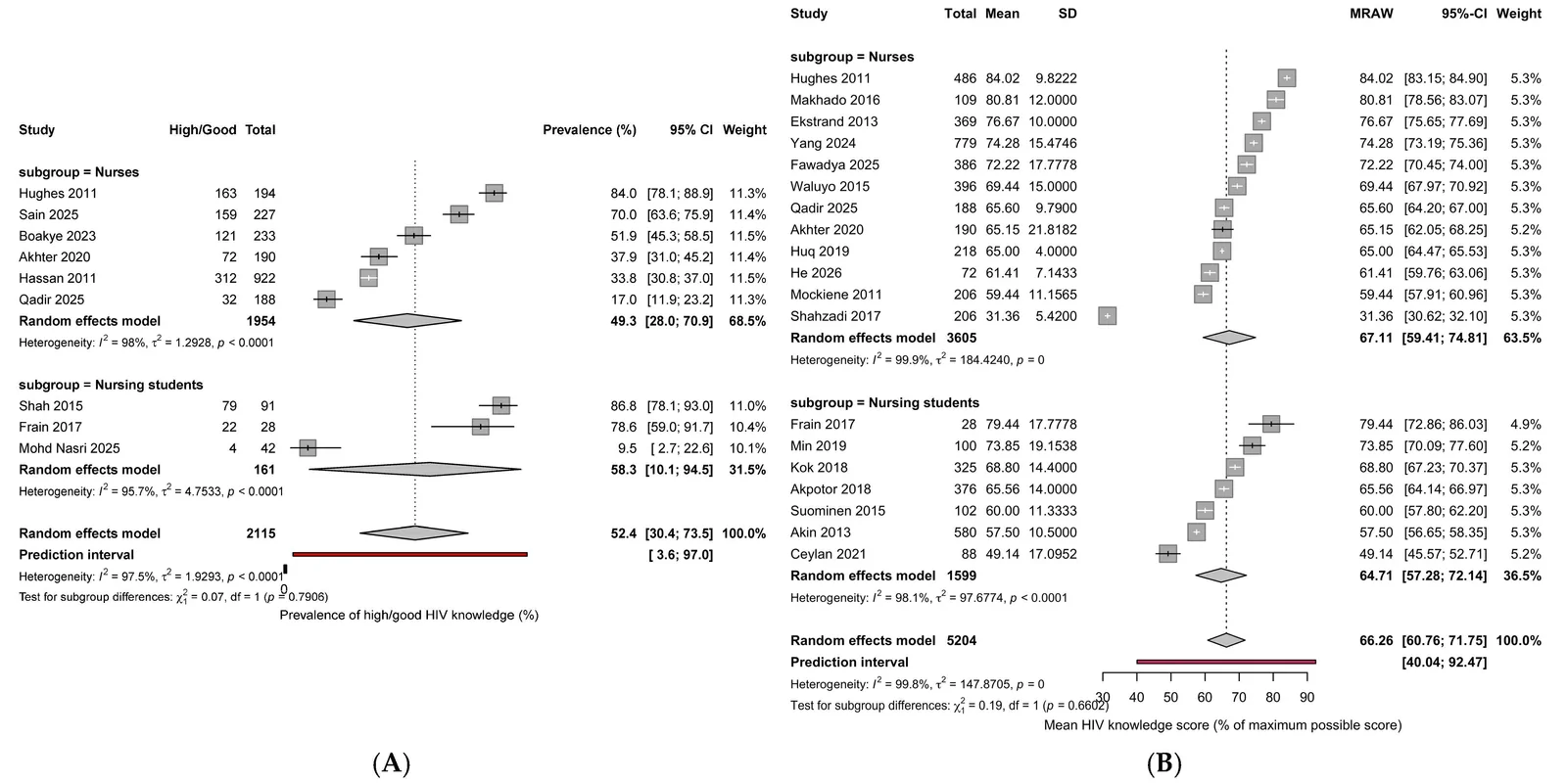
Meta-analysis of the Knowledge Domain: (**A**) Pooled High/Good Knowledge Prevalence; (**B**) Pooled Mean Knowledge Score. Panel (**A**) included: Hassan 2011 [46]; Hughes, 2011 [47]; Sain, 2025 [75]; Akhter, 2020 [66]; Boakye, 2023 [70]; Qadir, 2025 [74]; Shah, 2015 [58]; Frain, 2017 [4]; Mohd Nasri, 2025 [73]; Panel (**B**) included: Qadir, 2025 [74]; Akpotor, 2018 [62]; Waluyo, 2015 [60]; Ekstrand, 2013 [54]; Shahzadi, 2017 [61]; Mockiene, 2011 [48]; He, 2026 [76]; Yang, 2024 [6]; Hughes, 2011 [47]; Makhado, 2016 [35]; Fawadya, 2025 [72]; Huq, 2019 [63]; Frain, 2017 [4]; Min, 2019 [65]; Kok, 2018 [64]; Suominen, 2015 [59]; Akin, 2013 [53]; Ceylan, 2021 [67]; Akhter, 2020 [66].

**Figure 3:**
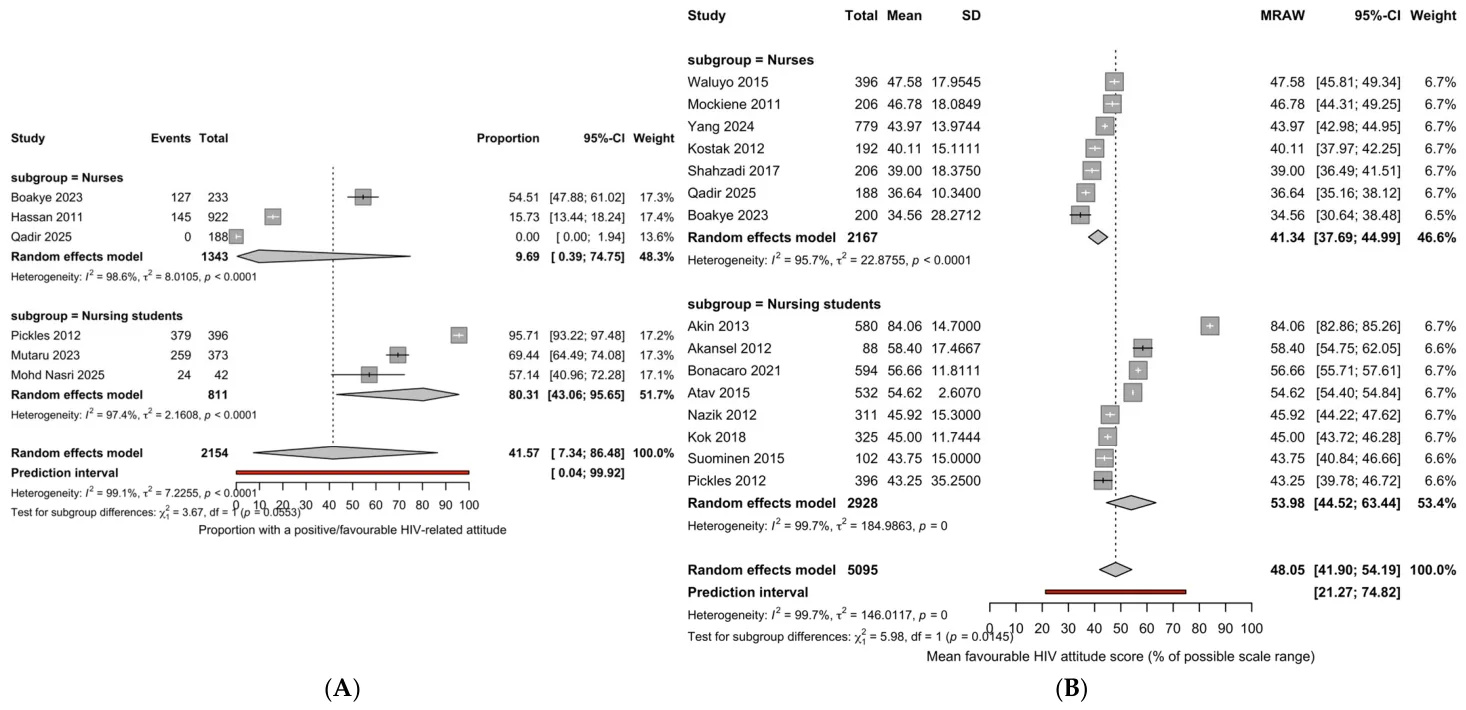
Meta-analysis of the Attitude Domain: (**A**) Pooled Positive/Favorable Attitude Prevalence; (**B**) Pooled Mean Favorable Attitude Score. Panel (**A**) included: Boakye, 2023 [70]; Hassan, 2011 [46]; Qadir, 2025 [74]; Mutaru, 2023 [71]; Pickles, 2012 [52]; and Mohd Nasri, 2025 [73]. Panel (**B**) included: Waluyo, 2015 [60]; Mockiene, 2011 [48]; Yang, 2024 [6]; Kostak, 2012 [50]; Shahzadi, 2017 [61]; Qadir, 2025 [74]; Boakye, 2023 [70]; Akin, 2013 [53]; Akansel, 2012 [49]; Bonacaro, 2021 [68]; Atav, 2015 [57]; Nazik, 2012 [51]; Kok, 2018 [64]; Suominen, 2015 [59]; and Pickles, 2012 [52].

**Figure 4:**
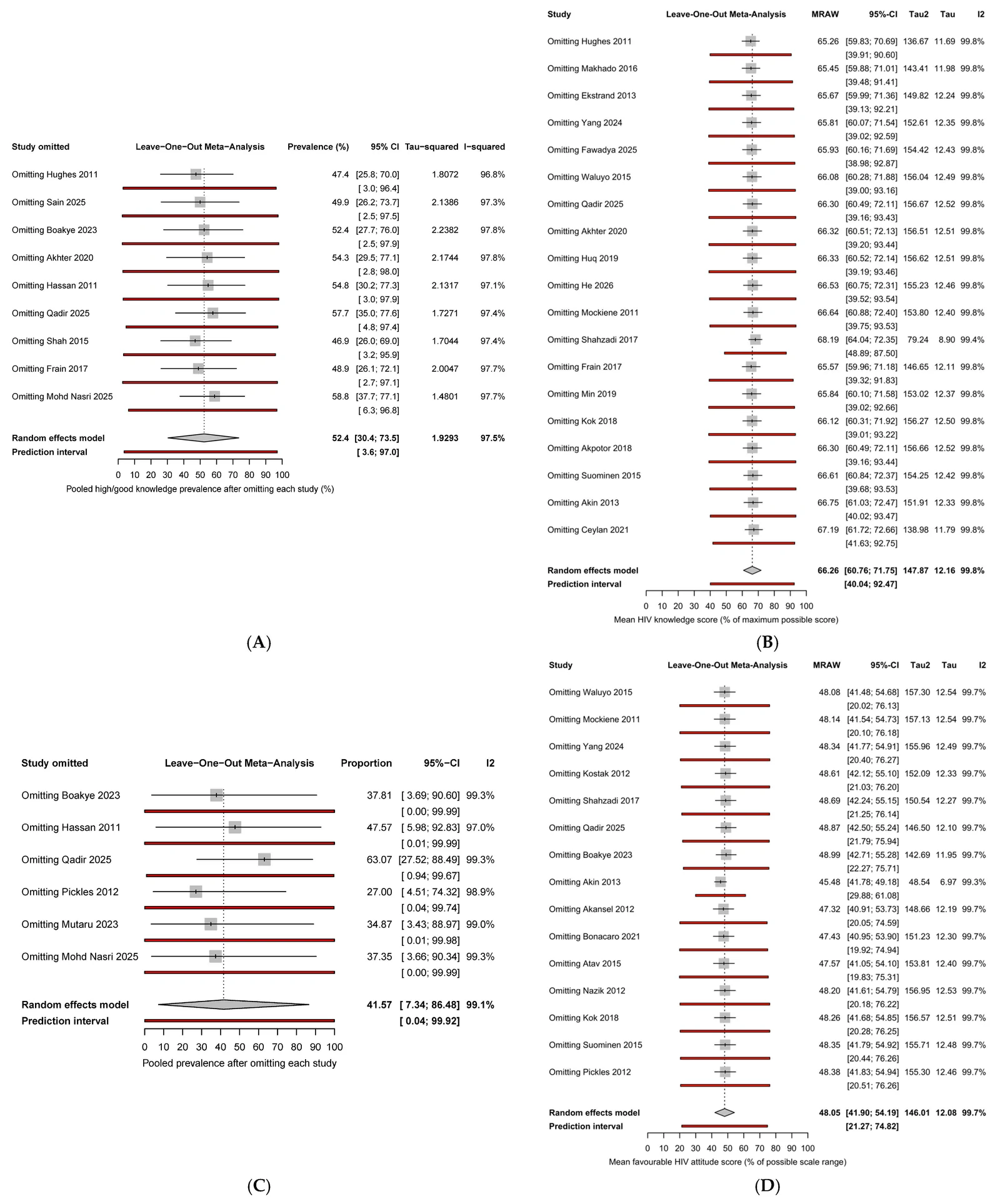
Leave-one-out sensitivity analysis: (**A**) Pooled High/Good Knowledge Prevalence; (**B**) Pooled Mean Knowledge Score; (**C**) Pooled Positive/Favorable Attitude Prevalence; (**D**) Pooled Mean Favorable Attitude Score. Panel (**A**) included: Hughes, 2011 [47]; Sain, 2025 [75]; Akhter, 2020 [66]; Boakye, 2023 [70]; Hassan, 2011 [46]; Qadir, 2025 [74]; Shah, 2015 [58]; Frain, 2017 [4]; Mohd Nasri, 2025 [73]; Panel (**B**) included: Qadir, 2025 [74]; Akpotor, 2018 [62]; Waluyo, 2015 [60]; Ekstrand, 2013 [54]; Shahzadi, 2017 [61]; Mockiene, 2011 [48]; He, 2026 [76]; Yang, 2024 [6]; Hughes, 2011 [47]; Akhter, 2020 [66]; Makhado, 2016 [35]; Fawadya, 2025 [72]; Huq, 2019 [63]; Frain, 2017 [4]; Min, 2019 [65]; Kok, 2018 [64]; Suominen, 2015 [59]; Akin, 2013 [53]; Ceylan, 2021 [67]. Panel (**C**) included: Boakye, 2023 [70]; Hassan, 2011 [46]; Qadir, 2025 [74]; Mutaru, 2023 [71]; Pickles, 2012 [52]; and Mohd Nasri, 2025 [73]. Panel (**D**) included: Waluyo, 2015 [60]; Mockiene, 2011 [48]; Yang, 2024 [6]; Kostak, 2012 [50]; Shahzadi, 2017 [61]; Qadir, 2025 [74]; Boakye, 2023 [70]; Akin, 2013 [53]; Akansel, 2012 [49]; Bonacaro, 2021 [68]; Atav, 2015 [57]; Nazik, 2012 [51]; Kok, 2018 [64]; Suominen, 2015 [59]; and Pickles, 2012 [52].

**Figure 5:**
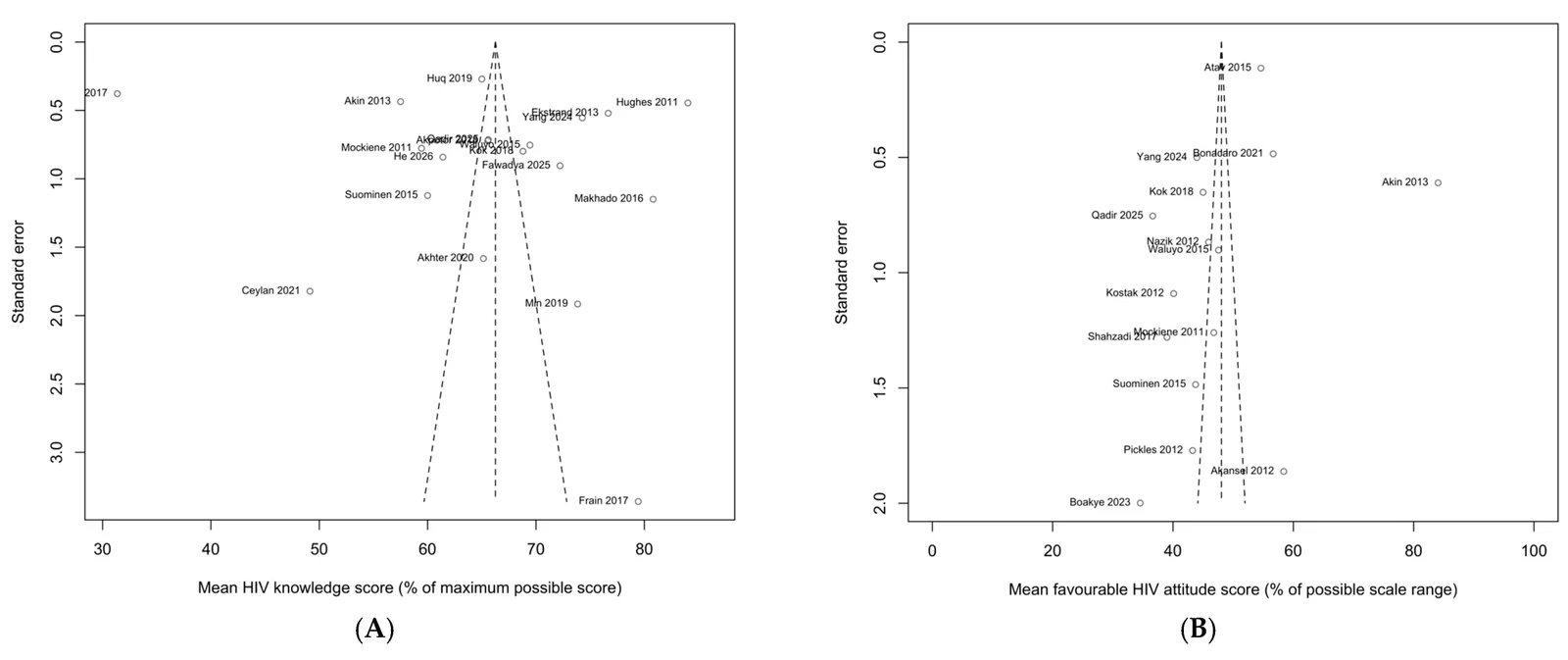
Publication bias assessment: (**A**) Pooled Mean Knowledge Score; (**B**) Pooled Mean HIV Attitude Score. Panel (**A**) included: Qadir, 2025 [74]; Akpotor, 2018 [62]; Waluyo, 2015 [60]; Ekstrand, 2013 [54]; Shahzadi, 2017 [61]; Mockiene, 2011 [48]; He, 2026 [76]; Yang, 2024 [6]; Hughes, 2011 [47]; Makhado, 2016 [35]; Fawadya, 2025 [72]; Huq, 2019 [63]; Frain, 2017 [4]; Min, 2019 [65]; Kok, 2018 [64]; Suominen, 2015 [59]; Akin, 2013 [53]; Ceylan, 2021 [67]; Akhter, 2020 [66]. Panel (**B**) included: Waluyo, 2015 [60]; Mockiene, 2011 [48]; Yang, 2024 [6]; Kostak, 2012 [50]; Shahzadi, 2017 [61]; Qadir, 2025 [74]; Boakye, 2023 [70]; Akin, 2013 [53]; Akansel, 2012 [49]; Bonacaro, 2021 [68]; Atav, 2015 [57]; Nazik, 2012 [51]; Kok, 2018 [64]; Suominen, 2015 [59]; and Pickles, 2012 [52].

**Table 1:** Systematic search strategies by database, date, and results.

Database	Search Strategy (Abbreviated)	Date Searched	Records Retrieved	Filters Applied
PubMed	(nurs*[tiab] OR nurse[MeSH] OR nursing staff[MeSH] OR nursing student*[tiab] OR nurse practitioner*[tiab] OR midwife*[tiab]) AND (“HIV”[MeSH] OR HIV[tiab] OR “human immunodeficiency virus”[tiab] OR AIDS[tiab] OR acquired immunodeficiency syndrome[tiab]) AND (knowledge[tiab] OR attitude*[tiab] OR practice*[tiab] OR stigma[tiab] OR discrimination[tiab] OR willingness[tiab] OR perception*[tiab] OR belief*[tiab] OR competency[tiab] OR confidence[tiab] OR preparedness[tiab] OR KAP[tiab]) AND (2011:2026[pdat])	May 2026	1989	English language, Humans, 2011–2026
Scopus	TITLE-ABS-KEY(nurs* OR “nursing student*” OR midwife*) AND TITLE-ABS-KEY(HIV OR AIDS OR “human immunodeficiency virus”) AND TITLE-ABS-KEY(knowledge OR attitude* OR practice* OR stigma OR discrimination OR willingness OR perception* OR preparedness OR competency)	May 2026	3575	English language, Article type, 2011–2026
PsycINFO	XB (DE “Nurses” OR DE “Registered Nurses” OR … TI(nurs* OR “nursing student*” OR midwi*)) AND XB (DE “HIV” OR TI(HIV OR AIDS OR …)) AND XB (DE “Knowledge” OR DE “Attitudes” OR … TI(knowledge OR attitude* OR practice* OR stigma OR stigmatiz* OR discriminat* OR belief* OR KAP)) [Date: January 2011–June 2026; Language: English]	May 2026	822	English language, 2011–2026
CINAHL Complete	XB (MH “Nurses+” OR MH “Nursing Students+” OR TI(nurs* OR midwi*)) AND XB (MH “HIV” OR TI(HIV OR AIDS)) AND XB (MH “Knowledge” OR TI(knowledge OR awareness)) AND XB (MH “Attitude+” OR MH “Social Stigma” OR TI(attitude* OR stigma OR willingness OR fear*)) AND XB (MH “Clinical Competence” OR TI(practice* OR competency OR preparedness)) [Date: January 2011–June 2026; Language: English]	May 2026	450	English language, 2011–2026
CINAHL Complete	XB (nurse OR nursing students) AND XB (HIV OR AIDS OR PrEP OR PEP) AND XB (knowledge OR Attitude OR Practice OR Stigma) [Date: January 2011–June 2026; Language: English, Scholarly/Peer reviewed]	July 2026	1098	English language, Scholarly/Peer reviewed, 2011–2026

Note: MeSH = Medical Subject Headings; DE = Descriptor (controlled vocabulary term).

**Table 2:** Characteristics of included studies in the systematic review and meta-analysis of nurses’ knowledge, attitudes and practices towards HIV.

First Author, Year	Country	WHO Region	Income Level	Study Design	Population Type	N	Assessment Instrument(s) & Methods	KAP Domains Analyzed
Hassan 2011 [46]	Jordan	Eastern Mediterranean	Upper-middle	Cross-sectional	Registered nurses	922	Self-administered Eckstein questionnaire; knowledge 33 items; attitude 0–175	Knowledge (% correct) Attitude (positive) Practice (refusal of care item)
Hughes 2011 [47]	United States	Americas	High	Cross-sectional	Registered nurses	194	HIV Knowledge Questionnaire-45, AIDS Attitude Scale	Knowledge (mean value, % high knowledge), attitude (mean)
Mockiene 2011 [48]	Lithuania	Europe	High	RCT (educational intervention)	Registered nurses	206 (baseline) 185 (follow-up)	Modified SUNY Buffalo AIDS Knowledge 33 items; attitude 35 items;	Knowledge (0–33) Attitude (1–5) (Baseline data)
Akansel 2012 [49]	Turkey	Europe	Upper-middle income	Cross-sectional	Nursing students	88 (first year undergraduate)	HIV knowledge AIDS Attitude Scale	Knowledge (% adequate level); attitude mean score
Kostak 2012 [50]	Turkey	Europe	Upper-middle income	Cross-sectional	Nurses	192	AIDS Attitude Scale	Attitude mean values
Nazik 2012 [51]	Turkey	Europe	Upper-middle income	Cross-sectional	Nursing students	468	AIDS Attitude Scale	Attitude mean values
Pickles 2012 [52]	Australia	Western Pacific Region	High	Cross-sectional	Nursing students	396	HIV knowledge Attitudes toward caring for PLHIV	Knowledge mean values; Attitude mean values
Akin 2013 [53]	Turkey	Europe	Upper-middle income	Cross-sectional	Nursing students	580	Attitudes toward caring for PLHIV	Attitude mean values
Ekstrand 2013 [54]	India	South-East Asia	Lower-middle	Cross-sectional	Nurses (extracted from mixed HCP sample)	369	Structured interview: HIV knowledge (0–15);	Knowledge (0–15) Practice: discriminatory intent items
Hamama, 2014 [55]	Russia	European	High	Cross-sectional	Nurses caring for PLHIV	69	AIDS Attitude Scale	Attitude mean values
Özakgül 2014 [56]	Turkey	Europe	Upper-middle	Cross-sectional	Nursing students (a) provided care to HIV patients (b) did not provide care	614	AIDS Attitude Scale	Attitude mean values
Atav 2015 [57]	Turkey and USA	mixed	mixed	Cross-sectional	Nursing students	370 Turkey and 241 USA	AIDS Attitude Scale	Attitude mean values (a) female; (b) male
Shah 2015 [58]	India	South-East Asia	Lower-middle	Interventional	Nursing students	91	HIV knowledge, attitudes toward caring for PLHIV	Knowledge (% high), and attitude (mean)
Suominen 2015 [59]	Russia	European	High	Cross-sectional	Nursing students	102	HIV knowledge, HIV attitude	HIV knowledge (mean score), HIV attitude (mean score)
Waluyo 2015 [60]	Indonesia	South-East Asia	Upper-middle	Cross-sectional	Registered nurses	396	AIDS Attitude Scale; HIV knowledge 18-item scale	Knowledge (mean); AAS continuous
Makhado 2016 [35]	South Africa	Africa	Upper-middle	Cross-sectional	Practicing Nurses	109	HIV Knowledge	HIV Knowledge mean score
Frain 2017 [4]	United States	Americas	High	Pre-post educational intervention	Undergraduate nursing students	28	HIV-KQ-18 (0–18); HIV/AIDS Questionnaire for Health Care Providers	Knowledge (HIV-KQ-18 pre/post)
Shahzadi 2017 [61]	Pakistan	Eastern Mediterranean	Lower-middle	Cross-sectional	Registered nurses (≥1 HIV patient cared for)	206	KAP Likert scales (knowledge 10 items; attitude 5 items; practice 10 items)	Knowledge (mean) Attitude (mean, % max) Practice (mean)
Akpotor 2018 [62]	Nigeria	Africa	Lower-middle	Cross-sectional survey	Nursing students (2nd–3rd year)	376	HIV knowledge 9 items	Knowledge (0–9)
Huq 2019 [63]	Barbados	Americas	High	Cross-sectional	Registered nurses	218	HIV knowledge and attitude questionnaire	HIV knowledge (mean score); HIV attitude (mean score)
Kok 2018 [64]	Turkey	Europe	Upper-middle income	Cross-sectional	Nursing students	580	HIV knowledge AIDS attitude scale	Mean values
Min 2019 [65]	Korea	Western Pacific	High	Cross-sectional	Nursing students	100	HIV knowledge scale	Mean values
Akhter 2020 [66]	Pakistan	Eastern Mediterranean	Lower-middle	Cross-sectional	Charge nurses	190	Adapted from AJN/Eckstein; knowledge; attitude (14-item Likert)	Knowledge (categories + mean) Attitude (item-level only) Practice (composite % + items)
Ceylan 2021 [67]	Turkey	Europe	Upper-middle income	Cross-sectional	Nursing students	88	AIDS knowledge scale	Mean values
Bonacaro 2021 [68]	Mixed	Europe	High	Cross-sectional	Nursing students	594	AIDS Attitude Scale	Mean values
Dlamini 2022 [69]	Eswatini	Africa	Lower-middle	Cross-sectional	Final-year nursing students	140	Adapted HIV/AIDS knowledge questionnaire	Knowledge (continuous mean)
Boakye 2023 [70]	Ghana	Africa	Lower-middle	Correlational cross-sectional	Registered nurses	247	48-item HIV/AIDS KAP questionnaire	Knowledge Attitude Practice (Category prevalences; Spearman correlations)
Mutaru 2023 [71]	Ghana	Africa	Lower-middle	Cross-sectional analytical	Trainee/student nurses	373	Adapted KAP questionnaire; knowledge 0–21; attitude 0–8; practice 0–10;	Knowledge Attitude Practice (Category prevalences)
Yang 2024 [6]	China	Western Pacific	Upper-middle	Quasi-experimental (AWWI intervention)	Hospital nurses	779	HIV/AIDS knowledge questionnaire (0–12); general prejudicial attitude scale (0–40); avoidance behaviour scale	Knowledge; Attitude Avoidance behavior (Practice-proxy) (Baseline data)
Fawadya 2025 [72]	Indonesia	South-East Asia	Upper-middle	Cross-sectional	Nurses	386	18 item HIV knowledge questionnaire HIV-KQ-18	Mean values
Mohd Nasri 2025 [73]	Malaysia	Western Pacific	Upper-middle	Cross-sectional	Nursing students	42	HIV knowledge and AIDS Attitude scale	High, moderate and low knowledge (%), positive and negative attitude (%)
Qadir 2025 [74]	Iraq	Eastern Mediterranean	Upper-middle	Cross-sectional	Registered nurses	188	Researcher-developed questionnaire; knowledge 0–100; attitude 0–100 (standardized)	Knowledge (% max) Attitude (% max, higher = favorable) + category prevalences
Sain 2025 [75]	India	South-East Asia	Lower-middle	Interventional	Nurses	227	General HIV/AIDS knowledge with separate needle-stick HIV knowledge	High adequate and low level of knowledge
He 2026 [76]	Taiwan	Western Pacific	High	Mixed-methods RCT (experiential HIV self-testing)	Emergency nurses	72	HIV Knowledge Scale (0–28);	Knowledge (0–28) Knowledge (Baseline)

Abbreviations: AAS = AIDS Attitude Scale; AIDS = acquired immunodeficiency syndrome; HCP = healthcare professional; HIV = human immunodeficiency virus; HIV-KQ-18 = HIV Knowledge Questionnaire-18; KAP = knowledge, attitudes, and practices; N = sample size; PLHIV = people living with HIV; RCT = randomized controlled trial; WHO = World Health Organization. Income level classification: World Bank 2023/2024 criteria.

**Table 3:** Study-specific HIV-related clinical-practice indicators among nurses and nursing students.

Study ID	Events, *n*	Total, *n*	Description
Hassan 2011 [46] nurses	147	920	Single behavior: did not refuse direct care to a patient with AIDS
Akhter 2020 [66] nurses	162	190	Study-defined overall good practice
Boakye 2023 [70] nurses	128	228	Study-defined overall good practice
Mutaru 2023 [71] nursing students	215	373	Study-defined HIV/AIDS-prevention practice

**Table 4:** GRADE certainty of evidence assessment.

Outcome	k	Certainty	Reasons
(i) Proportion with high/good HIV knowledge	9	Very low	Risk of bias (−1): several contributing studies had non-probability/selected recruitment, incomplete response information, or measurement limitations.|Inconsistency (−2): I^2^ = 98% (considerable heterogeneity).|Imprecision (−1): pooled N = 2115 and/or wide 95% CI around the pooled estimate.|Publication bias: not assessable (k < 10 studies; Egger’s test not run).
(ii) Mean HIV knowledge score	19	Very low	Risk of bias (−1): multiple contributing studies used convenience or purposive samples, lacked formal sample-size justification or response information, or had incomplete psychometric reporting.|Inconsistency (−2): I^2^ = 100% (considerable heterogeneity). Publication bias suspected.
(iii) Proportion with favorable attitude toward PLHIV	6	Very low	Risk of bias (−1): the small evidence base included studies with sampling limitations and study-specific measurement/cut-off concerns.|Inconsistency (−2): I^2^ = 99% (considerable heterogeneity).|Imprecision (−1): pooled N = 2154 and/or wide 95% CI around the pooled estimate.|Publication bias: not assessable (k < 10 studies; Egger’s test not run).
(iv) Mean attitude score (reversed to favorable direction)	15	Very low	Risk of bias (−1): convenience/purposive recruitment and incomplete response reporting were common among contributing studies, with additional measurement uncertainty in some adapted or researcher-developed instruments.|Inconsistency (−2): I^2^ = 100% (considerable heterogeneity).|Imprecision (−1): pooled N = 5095 and/or wide 95% CI around the pooled estimate. Publication bias suspected.

## Data Availability

The original contributions presented in this study are included in the article. Further inquiries can be directed to the corresponding author Indira Karibayeva (ik01379@georgiasouthern.edu).

## Supplementary Materials

The following supporting information can be downloaded at: https://www.mdpi.com/article/10.3390/healthcare14193159/s1, Figure S1: Secondary meta-analysis of study-defined moderate-or-higher HIV-related knowledge prevalence; Table S1: Item-level JBI risk-of-bias assessment results.
