# Supplementary material for: General HIV-Related Knowledge and Attitudes Among Nurses and Nursing Students: A Systematic Review and Meta-Analysis with Narrative Synthesis of Clinical Practices (2011–2026)
Source: Healthcare (Basel). 2026 Sep 23;14(19):3159. doi: 10.3390/healthcare14193159 (PMC13665525; doi:10.3390/healthcare14193159)
Supplement: Supplementary file 1 [file healthcare-14-03159-s001.zip › Table S1.pdf]

Table S1. Item-level JBI risk-of-bias assessment results

Study	Q1	Q2	Q3	Q4	Q5	Q6	Q7	Q8	Q9	Key appraisal consideration
Hassan 2011 [46]	N	N	Y	Y	Y	Y	Y	Y	Y	Selective facility participation (20 of 87 hospital administrators agreed); nurse-level probability sampling was not demonstrated.
Hughes 2011 [47]	Y	Y	U	Y	Y	Y	Y	Y	U	National random sampling from professional membership lists; formal sample-size justification and non-response management were not clearly reported.
Mockiene 2011 [48]	Y	Y	U	Y	Y	Y	Y	Y	Y	Baseline observations were used; formal sample-size justification for the descriptive baseline estimate was not explicit.
Akansel 2012 [49]	Y	Y	U	Y	Y	Y	Y	Y	Y	Eligible first-year cohort was invited; formal sample-size justification was not explicit.
Kostak 2012 [50]	U	U	U	Y	Y	Y	Y	Y	U	Sampling frame, participant selection, sample-size justification, and response information were incompletely reported.
Nazik 2012 [51]	Y	N	U	Y	Y	Y	Y	Y	N	Attendance-based recruitment: 311 students present at the time of data collection were analyzed from a source population of 468; non-response was not otherwise addressed.
Pickles 2012 [52]	Y	N	U	Y	Y	Y	Y	Y	Y	Recruitment was based on attendance at a scheduled lecture rather than probability sampling; achieved response was otherwise high.
Akin 2013 [53]	Y	U	U	Y	Y	Y	Y	Y	U	Participant sampling, sample-size justification, and response information were not sufficiently clear in the report.
Ekstrand 2013 [54]	Y	N	U	Y	Y	Y	Y	Y	U	Hospital/department-assisted recruitment was non-probability; a response denominator was not clearly reported.
Hamama, 2014 [55]	Y	U	U	Y	Y	Y	Y	Y	Y	Sampling procedure and sample-size justification were incompletely reported; approximately 90% of approached nurses participated.
Özakgül 2014 [56]	Y	U	U	Y	Y	Y	Y	Y	U	Sampling procedure, sample-size justification, and response handling were incompletely reported.
Atav 2015 [57]	Y	U	U	Y	Y	Y	Y	Y	U	Sampling procedure, sample-size justification, and response handling were incompletely reported.
Shah 2014 [58]	Y	Y	U	Y	Y	Y	Y	Y	Y	Baseline class sample was nearly complete; formal sample-size justification was not explicit.
Suominen 2015 [59]	N	N	U	Y	Y	Y	Y	Y	Y	Three of ten nursing schools were purposively selected; students were not sampled probabilistically.
Waluyo 2015 [60]	N	N	Y	Y	Y	Y	Y	Y	U	Hospitals and nurses were recruited using convenience/quota procedures; a conventional response denominator was unclear.
Makhado 2016 [35]	Y	Y	U	Y	Y	U	Y	Y	Y	Stratified random sampling and high response were reported; validity evidence for the specific general HIV knowledge measure was incompletely documented.

Study	Q1	Q2	Q3	Q4	Q5	Q6	Q7	Q8	Q9	Key appraisal consideration
Frain 2017 [4]	Y	Y	U	Y	Y	Y	Y	Y	Y	Near-complete class participation; the small baseline sample had no formal descriptive sample-size justification.
Shahzadi 2017 [61]	N	N	U	Y	U	U	U	Y	U	Convenience sampling, incomplete questionnaires, and limited psychometric/scoring information created several appraisal uncertainties.
Akpotor 2018 [62]	Y	U	U	Y	Y	Y	Y	Y	Y	Study frame and achieved response were adequate; within-school sampling and sample-size justification were not fully reported.
Huq 2019 [63]	Y	Y	U	Y	Y	Y	Y	Y	Y	Systematic sampling was used and response was 79%; formal sample-size justification was not explicit.
Kok 2018 [64]	N	N	U	Y	Y	Y	Y	Y	U	Single-faculty volunteer sample; the denominator for response was not reported.
Min 2019 [65]	Y	N	U	Y	Y	U	U	Y	U	Single-university volunteer sample; sample-size justification and response denominator were not reported, and psychometric support for the 13-item knowledge measure was not reported in the article.
Akhter 2020 [66]	N	N	U	Y	Y	U	U	Y	U	Non-probability convenience sampling and limited reporting of measurement validity/reliability and response.
Ceylan 2021 [67]	Y	N	U	Y	Y	Y	Y	Y	U	Voluntary recruitment from a single course; response denominator and formal sample-size justification were not clearly reported.
Bonacaro 2022 [68]	Y	N	U	Y	Y	Y	Y	Y	U	Convenience multicenter student sample; response denominator was not reported.
Dlamini 2022 [69]	Y	N	Y	Y	Y	Y	Y	Y	Y	Convenience sampling; otherwise explicit sample-size planning, instrument reliability, and high achieved recruitment.
Boakye 2023 [70]	Y	Y	Y	Y	U	Y	Y	Y	U	Probability sampling and sample-size planning were reported; variable-specific missing denominators and response handling were incompletely reported.
Mutaru 2023 [71]	Y	Y	Y	Y	Y	Y	Y	Y	Y	Multistage stratified random sampling, explicit sample-size planning, complete response, and reliability reporting for adapted scales.
Yang 2024 [6]	N	N	U	Y	Y	Y	Y	Y	Y	Nursing units were purposively selected before allocation; baseline measurement and retention were otherwise well documented.
Fawadya 2025 [72]	Y	N	U	Y	Y	Y	Y	Y	U	Consecutive single-hospital sampling; response denominator and formal sample-size justification were not reported.
Mohd Nasri 2025 [73]	Y	Y	Y	Y	Y	Y	Y	Y	Y	Proportionate stratified random sampling, explicit sample-size calculation, complete response, and reliable measures.
Qadir 2025 [74]	Y	Y	Y	Y	Y	U	U	Y	Y	Census recruitment and good response; the researcher-developed measurement had limited psychometric support (attitude alpha approximately 0.60; knowledge reliability not reported).

Study	Q1	Q2	Q3	Q4	Q5	Q6	Q7	Q8	Q9	Key appraisal consideration
Sain 2025 [75]	Y	Y	Y	Y	U	U	U	Y	N	All nurses were invited, but response was 45.4%; item-level missingness and limited validation/reliability reporting affected measurement confidence.
He 2026 [76]	Y	N	U	Y	Y	Y	Y	Y	N	Purposive/volunteer recruitment with approximately 47.7% enrollment; validated baseline measures were consistently applied.
